# Efficacy of afoxolaner (NexGard®) in preventing the transmission of *Leishmania infantum* and *Dirofilaria immitis* to sheltered dogs in a highly endemic area

**DOI:** 10.1186/s13071-021-04883-3

**Published:** 2021-07-29

**Authors:** Rossella Panarese, Roberta Iatta, Jairo Alfonso Mendoza-Roldan, Andrea Zatelli, Frederic Beugnet, Domenico Otranto

**Affiliations:** 1 Department of Veterinary Medicine, University of Bari, Valenzano, Italy; 2 Boehringer Ingelheim Animal Health, Lyon, France; 3 Faculty of Veterinary Sciences, Bu-Ali Sina University, Hamedan, Iran

**Keywords:** Dirofilariosis, Leishmaniosis, Incidence, Insecticide, Afoxolaner, NexGard®, Chemoprophylaxis

## Abstract

**Background:**

*Leishmania infantum* and *Dirofilaria immitis* are among the most important canine vector-borne pathogens (CVBPs) of zoonotic concern in Europe. In endemic areas for both of these CVBPs, the use of systemic ectoparasiticides, such as afoxolaner (NexGard®; Boehringer Ingelheim Animal Health), may have the potential for controlling these infections. The aim of this study was to assess, for the first time, the insecticidal efficacy of NexGard® in decreasing the transmission of *D. immitis* and *L. infantum* to sheltered dogs living in a hyperendemic area, compared to the year before treatment, as well as its impact on the abundance of mosquito and sand fly populations.

**Methods:**

All dogs (*n* = 179) enrolled in the study were divided into two groups based on their infection status at enrollment: a non-infected group (G1) and an infected group (G2; infected with *D. immitis*, *L. infantum* or both). The study was conducted from March 2020 to March 2021. In order to exclude all animals infected with *L. infantum* and *D. immitis* before March 2020 (sampling time: T0), dogs in G1 were sampled in June (T1; i.e. T0 + 90 days) and in October 2020 (T2; i.e. T0 + 210 days). From March to September 2020, all animals (G1 and G2) were weighed and treated monthly with NexGard®. Animals in G1 were tested for the last time in March 2021 (T3; i.e. T0 + 330 days) for assessing post-treatment incidence rate of infection and prevention efficacy.

**Results:**

The post-treatment incidence of *D. immitis* was 3.7% (1/27; 95% confidence interval [CI]: 0.2–18.1) and that of *L. infantum* was 3.6% (3/83; 95% CI: 1.0–10.1). Considering the annual incidence in 2019 and 2020, the protective efficacy against *D. immitis* and *L. infantum* infections was 94.2 and 64%, respectively. Of the female mosquitoes collected (*n* = 146), only one pool out of 50 tested positive for *D. immitis* DNA, whereas out of 1252 female *Sergentomya minuta* specimens collected, only four tested positive for *L. infantum* (0.3%).

**Conclusions:**

Afoxolaner is efficacious in decreasing the rate of transmission of both *D. immitis* and *L. infantum*; however, comparison of the pre- and post-treatment period demonstrated that there was a significant difference only in the seasonal incidences of *D. immitis* infection. Preventive measures are recommended throughout the year in endemic areas to reduce the risk of pathogen transmission to animals and humans.

**Graphical abstract:**

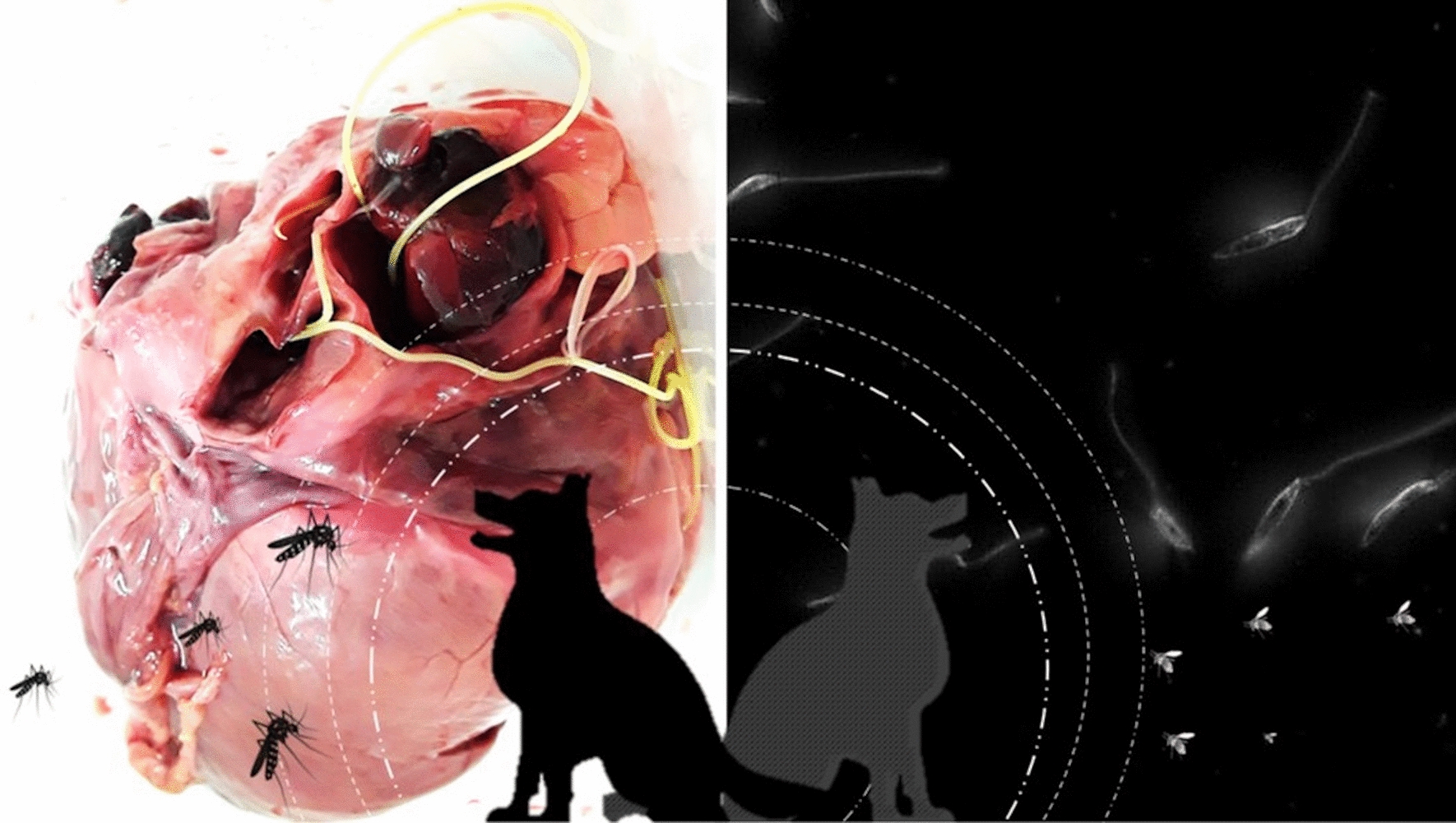

## Background

*Leishmania infantum* and *Dirofilaria* spp. are among the most important canine vector-borne pathogens (CVBPs) in Europe [1]. *Leishmania infantum*, a sand fly-transmitted protozoan, is the main causative agent of canine leishmaniosis (CanL) and of cutaneous and visceral leishmaniasis in humans [2]. The mosquito-transmitted nematode *Dirofilaria immitis* causes heartworm disease (HWD) in dogs and is also of zoonotic concern [1]. *Dirofilaria immitis* and *L. infantum* are widely distributed [1, 3], and the spread of these parasites is strictly related to the presence of infected dogs and vectors [3, 4]. *Dirofilaria immitis* infective stage larvae are transmitted by mosquito species of several genera (including *Aedes*, *Anopheles* and *Culex*) [5], whereas *L. infantum* infective promastigotes are vectored by phlebotomine sand fly species of the genus *Phlebotomus* in the Old World [3, 6]. Dogs living in areas of *D. immitis* and *L. infantum* endemicity are more susceptible to the infection during the activity season of their vectors, which is mostly related to the average seasonal temperature [7, 8].

In Italy, the epidemiology of both infections has been influenced by several factors, including vector distributions and chemoprophylactic treatments, which has over time resulted in a change in their original prevalence and distribution patterns throughout the country [9, 10]. An epidemiological survey conducted in a population of shelter dogs in Lecce province (Apulia region) revealed a high prevalence of *D. immitis* (53%) and *L. infantum* (58.1%) [11] and an annual incidence of 63.9 and 10%, respectively [11]. The circulation of *D. immitis* in this area has also been supported by the identification of two domestic cats infected with *D. immitis* and *D. repens*, respectively [12]. Given their high prevalence, the prevention of these parasitic infections is pivotal to reduce the high risk of infection in dogs, cats and humans [13, 14]. The current measures for *L. infantum* control, such as collars containing repellent insecticides, may be expanded by the use of isoxazoline systemic ectoparasiticides [15] by virtue of their insecticidal efficacy [16]. In a recent study, afoxolaner (Nexgard®; Boehringer Ingelheim Animal Health, Germany) has shown an insecticidal activity against *Aedes aegypti* [17] and *Phlebotomus perniciosus* [18]. It can therefore be hypothesized that regular monthly treatment of all dogs in a close environment could contribute to a decrease in the population of vectors and thus in the rate of pathogen transmission.

Taking all the data mentioned above into consideration, we conducted a field study to assess the decreased risk of *L. infantum* and *D. immitis* transmission based on treatment with afoxolaner (NexGard®) systemic insecticide on the vector population in a dog kennel where the vectors were collected, as well as the annual incidences of both CVBPs compared to the year before. In addition, the presence of *Dirofilaria* spp. and *L. infantum* was assessed in mosquito and sand fly populations trapped in the same enclosure.

## Methods

### Study design

The study was conducted from March 2020 to March 2021 in dogs living in a rescue shelter in the province of Lecce (40.419326°N, 18.165582°E; Apulian region, southern Italy) where CanL and HWD are highly endemic [11]. A total of 242 dogs living in the shelter were screened for entry into this non-controlled and non-blinded clinical field efficacy study. Animals were enrolled according to weight (≥ 2.0 kg), age (≥ 7 months old) and absence of major clinical conditions.

 Out of the 242 dogs living in the shelter, 179 were enrolled in the study and categorized based on their *Dirofilaria* spp. and *L. infantum* infection status. At enrollment (T0), blood sampling and clinical examination were conducted for each animal to establish the dog’s health status and to confirm the positivity or negativity to one or both infections. Due to the long pre-patent period of *D. immitis* infection, animals were further sampled at two time points, T1 (T0 + 90 days) and T2 (T0 + 210 days), in order to avoid positivity deriving from a previous infection (before March 2020). Subsequently, according to their infection status at T1, group 1 (G1) dogs were considered to be uninfected for *Dirofilaria* (G1-D) and/or *Leishmania* (G1-L), and group 2 (G2) dogs were considered to be infected by *D. immitis* and/or *L. infantum* (G2-D and G2-L, respectively) (Table 1). Thus, the animals in G2 were acting as continuous reservoirs for one or both pathogens. They were not subjected to any treatment against dirofilariosis or leishmaniosis except for medical necessity. The dogs did not receive any treatment with topical repellent insecticides, nor did they receive any heartworm preventative product.

**Table 1:** Number of dogs positive for *Dirofilaria immitis* and *Leishmania infantum* infections at three time points in 2020, prevalence of both infections in 2020 and number of new cases in March 2021

Time points^a^	*Leishmania infantum*				*Dirofilaria immitis*				
	IFAT-positive,* n*	IFAT-negative,* n*	Prevalence, *n*/total (%)	95% CI	Positive,* n*		Negative,* n*	Prevalence, *n*/total (%)	95% CI
					Knott’s test	SNAP test			
T0 (March 2020)	76	103 (G1-L)	76/179 (42.5)	35.4–49.8	68	115	63 (G1-D)	116^d^/179 (64.8)	57.6–71.4
T1 (June 2020)	86	83 (retained from 93^b,c^)	86/179 (48)	40.8–55.3	19	23	40	139/179 (77.7)	71.0–83.1
T2 (October 2020)	nt	nt	na	na	5	9	27 (retained from 30 ^b,c^)	149^d^/179 (83.2)	77.1–88.0
T3 (March 2021) or seasonal infection rate (incidence)	3	80	3/83 (3.6)	1.0–10.1	1	1	26	1/27 (3.7)	0.2–18.1

*CI* Confidence interval,* IFAT* immunofluorescence antibody test,*nt* not tested, *na* not applicable

^a^T0, enrollment (day 0 ); T1, T0 + 90 days); T2, T0 + 210 days; T0 + 330 days

^b^One and five dogs negative for *D. immitis* and *L. infantum*, respectively, were adopted after T2 and lost to further follow-up; therefore, they were not included in the evaluation of the post-treatment incidence in March 2021

^c^Three and five dogs negative for *D. immitis* and *L. infantum*, respectively, were included later in the study (T1 and T2) and their T3 (day + 330) was in April/June 2021; these dogs were therefore not included in the evaluation of the post-treatment incidence in March 2021

^d^The number of positive dogs increased by 1 based on quantitative PCR

From March to September 2020, all enrolled dogs (G1 + G2) were treated once a month with the oral-systemic insecticide NexGard® (afoxolaner: 2.7–6.9 mg/kg). Treatments were administrated during the vector season at day 0, +30, +60, +90, +120, +150 and +180 (± 7 days). Dogs were weighed before each treatment to determine the appropriate dosage, in accordance with the label.

### Efficacy assessment

The incidence for 2020 was calculated for dogs in G1 after the 2020 transmission season, during which they had been treated, and compared to that of the year before, during which there had been no treatment [19]. For this purpose, all dogs were tested at timepoints T1, T2 and T3 (T0 + 330 days).

The preventive efficacy of the monthly treatment was measured using this formula:

$$\begin{aligned} \% \,{\mkern 1mu} {\text{Decreased}}{\mkern 1mu} \,{\text{risk}}\,{\mkern 1mu} {\text{of}}\,{\mkern 1mu} {\text{transmission}} =\; & ({\text{incidence}}{\mkern 1mu} \,{\text{pre-treatment}} - {\text{incidence}}{\mkern 1mu} \,{\text{post-treatment}} \\ & /{\text{incidence}}{\mkern 1mu} \,{\text{pre-treatment}}) \times 100. \\ \end{aligned}$$

*Dirofilaria immitis* and *L. infantum* infections were studied separately; therefore, some dogs could be G1-D negative for *Dirofilaria* and G2-L positive for *Leishmania*, and vice versa. The analysis was performed with regard to G1-D and G1-L, corresponding to a treatment effect on different vectors, i.e. mosquitoes or sand flies.

### Blood sampling and diagnostic procedures

Whole blood (5 ml) from each dog was collected in an EDTA tube (2.5 ml) and in a tube containing a clot activator (2.5 ml), respectively. An aliquot (1 ml) of whole blood was processed using a modified Knott’s test to morphologically identify and determine the number of circulating microfilariae (mfs), as previously described [11]. A second aliquot of blood (100 µl) was processed by duplex real-time PCR (qPCR) to detect and identify *Dirofilaria* spp. [20]. Dog serum samples were also tested for the detection of the *D. immitis* female antigen using a commercial immunochromatographic assay (SNAP® 4Dx Plus test; (IDEXX, Westbrook, ME, USA), according to the manufacturer’s instructions. Serum samples were also tested for anti-*L. infantum* antibodies with a slightly modified immunofluorescence antibody test (IFAT) protocol, as previously described [21].

### Insect collections and infection rates

From May to November 2020, mosquito and sand fly specimens were collected in the dog shelter. Samplings were performed twice a month between 05:00 h and 08:00 h for both dipterans. Active adult mosquitoes were collected using two CO_2_-baited CDC light traps (Centers for Disease Control and Prevention, Atlanta, GA, USA), one gravid *Aedes* trap (GAT; BG-GAT; Biogents, Regensburg, Germany), one BG-sentinel-2 mosquito traps (Biogents) and one aspirator (InsectaVac Aspirator; BioQuip Products, Compton, CA, USA). Mosquito collections were carried out next to the dog cages or nearby stagnant water [22], and all captured mosquitoes were refrigerated until identification was made using morphological keys [23, 24]. Sand flies were collected using 64 sticky traps (white paper sheets coated with Castor oil [dimensions: 21.0 × 29.7 cm] covering a surface area of up to 4 m^2^) for each sampling and two CDC light traps [25]. Sand fly collections were carried out until their total disappearance (i.e. three consecutive negative collections). All specimens were stored in labeled glass vials containing 70% ethanol and then morphologically identified using taxonomic keys and descriptions [25, 26].

Pools of a maximum of ten specimens of mosquitoes or sand flies were tested by qPCR as described in section Molecular diagnosis. These pools were made based on specific criteria: species, site of the collection and collection date. The minimum infection rates (MIRs) were calculated using the standard formula for mosquito pools, as previously described [22]. The estimated rate of infection (ERI), which is adjusted for pooled samples, was calculated using the formula: ERI = 1 −  (1 − *x*/*m*)^1/*k*^, where *x* is the number of positive pools, *m* is the number of examined pools and *k* is the average number of specimens in each pool [27].

### Molecular diagnosis

Genomic DNA was extracted from blood and/or mfs as well as from pools of sand flies and mosquitoes (i.e. abdomen and thorax samples) using the GenUP™ Blood DNA Kit (Biotechrabbit GmbH, Berline, Germany) and the phenol/chloroform extraction method followed by ethanol precipitation, respectively [11, 28, 29]. Heads and the last segments of phlebotomine sand flies were previously removed for morphological identification. All blood and mosquito DNA samples were tested by qPCR, using two species-specific primer sets targeting partial cytochrome oxidase subunit 1 (*cox*1) for *D. immitis* and the second internal transcribed spacer-2 (ITS-2) of nuclear ribosomal DNA for *D. repens*, as previously described [20]. All sand fly samples were tested by duplex real-time PCR (dqPCR) for the detection of *Leishmania* spp. as previously described [30]. Approximately 100 ng of gDNA (except for the no-template control) was added to each dqPCR run. All DNA samples were tested in duplicate, and positive and negative controls were included in each qPCR run.

### Meteorological data

From May to November 2020, data on mean environmental temperature (°C), relative humidity (RH, %), monthly rainfall and wind speed were acquired from the climatological database of the “Agenzia Regionale per la Prevenzione e la Protezione dell’Ambiente” of Apulia Region. For the study site, relevant data from the nearest meteorological station were used for further analyses, such as correlating the meteorological data with the number of mosquitoes and sand flies caught each month.

### Data and statistical analyses

Data on the incidence of dirofilariosis and leishmaniosis in dogs and mosquito/sand fly populations were recorded in an Excel (Microsoft Corp., Redmond, WA, USA) spreadsheet and analyzed by Quantitative Parasitology 3.0. software in the subsequent statistical analyses [31].

The criterion followed was the seroconversion or PCR positivity observed in subgroups G1-D and G1-L. The association between the category variables in the dog population (i.e. sex, age, weight, entrance date in the dog shelter), in mosquitoes and sand flies collected (i.e. number of female specimens for each mosquito and sand fly species) and the positive results to *Dirofilaria* spp. and *L. infantum* were analyzed using contingency tables and Pearson’s Chi-squared test (*χ*^2^) values were calculated. Results were considered statistically significant if *P* < 0.05.

## Results

The dogs in G1 which subsequently tested positive for *L. infantum* at T1 (*n* = 10) were considered to be infected before the sand fly season; therefore, the number of *L. infantum*-negative dogs (G1-L) in G1 was 83 (Table 1). The dogs in G1 which were subsequently diagnosed to be positive for *D. immitis* at T1 and T2 (*n* = 33) were considered to have been infected before the mosquito season, resulting in a total of 27 negative animals (G1-D) (Table 1). From group G1-D, in March 2021 (T3), one dog tested positive for *D. immitis* according to Knott’s test, the SNAP 4Dx Plus test and qPCR, indicating a single infection during the 2020 season. Therefore, the observed incidence was 3.7% (1/27; 95% confidence interval: 0.2–18.1) (Table 1). Compared to the previous year’s incidence of 63.9% (39/61 dogs infected) [19], the efficacy of the systemic insecticide in reducing the transmission of *D. immitis* by mosquitoes was 94.2%, with a statistically significant difference in incidence between the 2 years of (*χ*^2^ = 27.38, *df* = 1, *P* < 0.0001).

From group G1-L, in March 2021 (T3), three dogs tested positive for *L. infantum*, giving an incidence of 3.6% (3/83; 95% CI: 1.0–10.1) (Table 1). Compared to the previous year incidence of 10% (7/70 dogs seroconverted in 2019) [19], the treatment efficacy in reducing the transmission of *L. infantum* by sand flies was 64%; however, the difference was not statistically significant (*χ*^2^ = 2.72, *df* = 1, *P* = 0.098).

The mosquitoes collected during the whole sampling period (*n* = 219) belonged to three different genera and six species (Table 2). Of the females collected (*n* = 146), only 16 were engorged (7 *Aedes albopictus*, 4 *Aedes caspius*, 4 *Culex pipiens* and *Culiseta annulata*). Among all the mosquito species collected, *C. pipiens* was the most prevalent species (*n* = 97, 44.3%). In 2019, 208 females were collected during the same season period, a decrease of 29.8% [11]. The number of female mosquitoes collected for each species in 2019 is reported in Table 3. The mean number of mosquito specimens for each trap type was 1.5 for BG-sentinel 2 trap, 13.5 for CDC-light trap and 4.5 for the GAT.

**Table 2:** Number of female mosquito specimens and positive pools molecularly tested for *Dirofilaria immitis* along with the average number of mosquitoes per pool, minimal infection rate and percentage of estimated rate of infection

Mosquito species	No. of mosquitoes	No. of females	No. of pools positive/tested	Mean no. of mosquitoes per pool	MIR (/1000)	ERI (%)
*Aedes caspius*	66	66	1/14	4.7	15.2	1.6
*Culex pipiens*	97	41	0/14	2.9	–	–
*Aedes albopictus*	28	16	0/10	1.6	–	–
*Aedes detritus*	11	10	0/3	3.3	–	–
*Culiseta annulata*	11	9	0/4	2.25	–	–
*Aedes berlandi*	2	2	0/2	1	–	–
*Aedes* spp.^a^	4	2	0/3	1	–	–
Total	219	146	1/50	2.4	6.8	0.8

*ERI* Estimated rate of infection,* MIR* minimal infection rate

^a^Damaged mosquito specimens morphologically identified at the genus level only

**Table 3:** Number of female mosquito specimens collected in 2019 in the dog shelter and positive pools molecularly tested for *Dirofilaria immitis* along with the average number of mosquitoes per pool, MIR and percentage of ERI

Mosquito species	No. of mosquitoes	No. of females	No. of pools positive/tested	Mean no. of mosquitoes per pool	MIR (/1000)	ERI (%)
*Aedes caspius*	131	129	3/15	8.6	23.3	3
*Culex pipiens*	43	37	0/11	3.4	–	–
*Aedes albopictus*	45	36	1/7	5.2	27.8	3
*Culiseta annulata*	1	1	0/1	1	–	–
*Culiseta longiareolata*	1	1	0/1	1	–	–
*Coquillettidia richiardii*	2	2	0/1	2	–	–
*Aedes detritus*	1	1	0/1	1	–	–
*Aedes mariae*	1	1	0/1	1	–	–
Total	225	208	4/38	5.5	19.2	2

Out of 50 mosquito pools, one (containing three *A. caspius* females) tested positive for *D. immitis* DNA, with an overall MIR of 6.8/1000. The overall ERI (i.e. the probability of a single positive mosquito specimen) was 0.8%. The MIR and ERI for each species separately are reported in Table 2, as well as the number of specimens for each mosquito species. In 2019, four pools out of 38 were PCR positive for *Dirofilaria*, although the difference between the 2019 and 2020 mosquito collections was not statistically significant (*χ*^2^ = 2.93, *df* = 1, *P* = 0.087).

A total of 2306 phlebotomine sand flies (2138 *Sergentomya minuta* and 168 *P. perniciosus*) were collected, of which 1281 were females (1252 *S. minuta* and 29 *P. perniciosus*). Four *S. minuta* females tested positive for *L. infantum* (0.3%). The mean monthly meteorological values obtained for the area of the dog shelter were: 21.8 °C; 70.5% RH, mean rainfall of 0.02 mm and mean wind speed of 2.04 m/s.

## Discussion

The results of this study suggest that the monthly administration of afoxolaner (NexGard®) to sheltered dogs in an endemic area for dirofilariosis and leishmaniosis is efficacious in terms of decreasing the rate of transmission of both *D. immitis* and *L. infantum*. The seasonal incidence for *D. immitis* infection observed in 2019 (63.9%) and 2020 (3.7%) are significantly different. In order to avoid false negative dogs due to a hard diagnosis of *D. immitis*, for both incidence evaluations we employed several diagnostic tools, and the overall positivity was considered to be the final incidence 2019/2020 [32]. We adopted the classical 5% error threshold and found that no other comparisons were significant; nevertheless, with the multiple factors involved and the high variability, we may consider the risk of error alpha to be 10% (*P* = 0.1). In that case, *Dirofilaria* and *Leishmania* infection incidence values differ between the pre- and post-treatment periods, as do the number of mosquito pools that tested positive for *D. immitis*. The observed result is not due to any repellent effect of NexGard®, but most likely due to the insecticidal activity of afoxolaner contributing not only to a decrease in density of vectors but also to a reduction in the risk of infecting bites [17]. Both female mosquitoes and sand flies need a few days to digest their blood meal and lay eggs before a new meal [16]. During this period of 3–5 days, the majority of mosquitoes and sand flies that have bitten dogs treated with afoxolaner die [17, 18, 33].

Mosquito and sand fly females that feed on a treated and infected animal (G2) will not transmit any pathogens due to their death after the blood meal as well as to the longer developmental times required by *Dirofilaria* spp. and *L. infantum* inside the vector [26, 34]. Therefore, afoxolaner may reduce the subsequent transmission of *Dirofilaria* spp. and *L. infantum* since their development requires more days than the length of survival of the vector [14].

Under field conditions, only repellent pyrethroids (e.g. deltamethrin, flumethrin, permethrin) have been tested to assess their preventive efficacy against CVBDs (e.g. [35, 36]). The decreased risk of *L. infantum* transmission addressed by repellents, with formulations containing permethrin or flumethrin, in field studies is greater (from 88.9 to 100%) than that herein reported for CanL using systemic insecticides [37–39]. Since data on the prevention of HWD infection through a repellent or a systemic insecticide in field studies are not available in the literature, a comparison of their efficacy is not possible. To date, afoxolaner has been shown to be efficacious in preventing the transmission of *Babesia canis* [40], as an effect of the induced rapid mortality of its vector *Dermacentor reticulatus* and the longer transmission time of this protozoan (up to 72 h after tick attachment). In our study, the activity of afoxolaner for the prevention of CanL and HWD is not related to the time of transmission of *Dirofilaria* spp. or *L. infantum* (4–5 min), but to a vector killing effect between two consecutive bites [16, 41].

The low number of female mosquitoes collected (*n* = 146) represents a 30% decrease compared to the number collected the previous year (*n* = 208; Table 3); however, the limited sampling results prevent any definitive conclusions to be drawn on the abundance of female mosquitoes over the 2 years of the study. Accordingly, the overall MIR (6.8/1000) was lower than that recorded in 2019 (19.2/1000), with only one pool of *A. caspius* testing positive for *D. immitis* DNA. Moreover, the relative decrease in the number of *A. caspius* and relative increase in the number of *C. pipiens* collected compared to the year before could be due to the average temperature of the 2020 season being lower than that of the previous year [11], as well as to a potential different susceptibility of the two mosquito species to the insecticide used in the study. However, the impact of the insecticide on the density of the mosquito population should be further investigated. The higher occurrence of *S. minuta*, the sand fly species with herpetophilic attitude, than *P. perniciosus,* with mostly zoophilic behavior, could be related to the effect of afoxolaner treatment on the studied dog population. However, despite their different blood-feeding behaviors, four *S. minuta* tested positive for *L. infantum*, suggesting a putative role of this sand fly species in the transmission of this pathogen [42, 43] and a different susceptibility to afoxolaner compared to *P. perniciosus* [19, 44]. Both the above assumptions need to be further investigated.

## Conclusions

The protection of dogs from infective bites of mosquito and sand fly vectors reduces their capacity to act as reservoirs of pathogens. Based on the One Health approach, dogs in endemic areas with a high risk of VBP exposure should be treated to decrease/prevent the risk of infection as well as the spread of these parasites to other animals and humans living in the same geographical area. Afoxolaner is efficacious in decreasing the rate of transmission of both *D. immitis* and *L. infantum*, although we found a significant difference between the 2 years of study only in the seasonal incidences of *D. immitis* infection. Our study is the first demonstration that systemic insecticides without repellent activity may play a role in decreasing the risk of pathogen transmission by mosquitoes and sand flies.

## Data Availability

The datasets used and/or analysed during the current study are available from the corresponding author on reasonable request.

## Abbreviations

CVBPS: Canine vector-borne pathogens
ERI: Estimated rate of infection
G1: Group 1
G2: Group 2
GAT: Gravid *Aedes* trap
HWD: Heartworm disease
IFAT: Immunofluorescence antibody test
MIR: Minimum infection rate
